# Somatic mutation driven codon transition bias in human cancer

**DOI:** 10.1038/s41598-017-14543-1

**Published:** 2017-10-27

**Authors:** Hyeonju Son, Hyundeok Kang, Hyun Seok Kim, Sangwoo Kim

**Affiliations:** Severance Biomedical Science Institute, Brain Korea 21 PLUS Project for Medical Sciences, Yonsei University College of Medicine, Seoul, 03722 South Korea

## Abstract

Accumulation of DNA mutations alters amino acid sequence in the key domains of oncoproteins, leading to cellular malignant transformation. Due to redundancy of the genetic code, the same amino acid alteration can be achieved by multiple distinct genetic mutations, which are considered functionally identical and not actively distinguished in the current cancer genome research. For the first time, we analyzed the distribution of codon level transitions acquired by somatic mutations in human cancers. By analyzing the ~2.5 million nonsynonymous somatic single nucleotide variations (SNVs) found in the COSMIC database, we found 41 recurrent amino acid alterations whose DNA changes are significantly biased toward a specific codon transition. Additional analyses partially identified functional discrepancies between the favored and avoided codon transitions in terms of mutational process, codon usage, alternative splicing, and mRNA stability.

## Introduction

Cancer is a genetic disease caused by a single or a few catastrophic somatic mutations that are responsible for cellular transformation, with accompanying many passenger mutations^1^. Many of the identified causal genetic changes are a substitution of one base pair in DNA (single nucleotide variation, SNV) that alters an amino acid in the corresponding codon (nonsynonymous change). The mutant protein, in turn, may initiate the cascades of downstream signaling (e.g., cell proliferation and inhibition of apoptosis) and eventually transforms the cellular phenotype from normal to malignant. In this context, the semantic basis of the entire event is the change of amino acids, not nucleotides, because different nonsynonymous SNVs are translated to the identical protein level alteration, thereby leading to the same functional impact. With the additional difficulty in locating the exact genomic coordinates under the continuing updates of the human reference genome, cancer-associated variants are usually represented in terms of the amino acid alteration.

Nonetheless, the redundancy of the genetic code not only causes the amino acid changes but also, along with its observational frequency, codon usage can have other functional effects beyond altering translation efficiency^2^. So far, many studies have shown that multiple synonym codons (a set of codons that are translated into the same amino acid) have different efficiency regarding the translation process on the ribosome including the speed of translation and folding accuracy^3^. Thus, the use of one codon, instead of the others, may offer a slight advantage or disadvantage over other competitors for a limited resource, especially in some model organisms^4–7^. One working hypothesis is that when mutations are accumulated for a long period, entities may have a significant fitness advantage over others, which can results in a deviation among synonym codons^8–10^. Conversely, numerical representation of this deviation, such as relative synonymous codon usage (RSCU)^11^ enables researchers to infer whether there has been a codon level selection. (see Supplementary Table S1 for the RSCU of human cells) Even without identifying the exact molecular process, it is known that the synonym codon usage bias (CUB) is common in many species^12^.

Just as the model organisms and systems for CUB analysis, the tumor environment can be seen as a microcosm of an ecosystem under rapid natural selection^13,14^ because it satisfies the essential prerequisites of evolution: First, genetic variation occurs with an increased frequency due to the uncontrolled proliferation and the compromised DNA integrity. Second, cells with different genetic traits (e.g., growth factors or cell cycle regulators) acquire different proliferation and death rates^10,15^. Third, the acquired mutant genotypes are efficiently inherited by descendants via clonal expansion. Moreover, cancer cells are usually under harsh environmental pressure such as hypoxia^16^, glucose starvation^17^, or even physical forces^18^ to expedite the evolutionary process. This analogy leads us to an intriguing question regarding the CUB in cancer cells: is there a favored codon used in cancer mutations? Somatic mutations are believed to occur in mostly random manner across the genome; therefore, a biased frequency among mutant synonym codons can be a putative sign for the codon level selection in cancer cells along with the conventionally considered external factors such as environmental effects and drug treatment.

**Figure 1:**
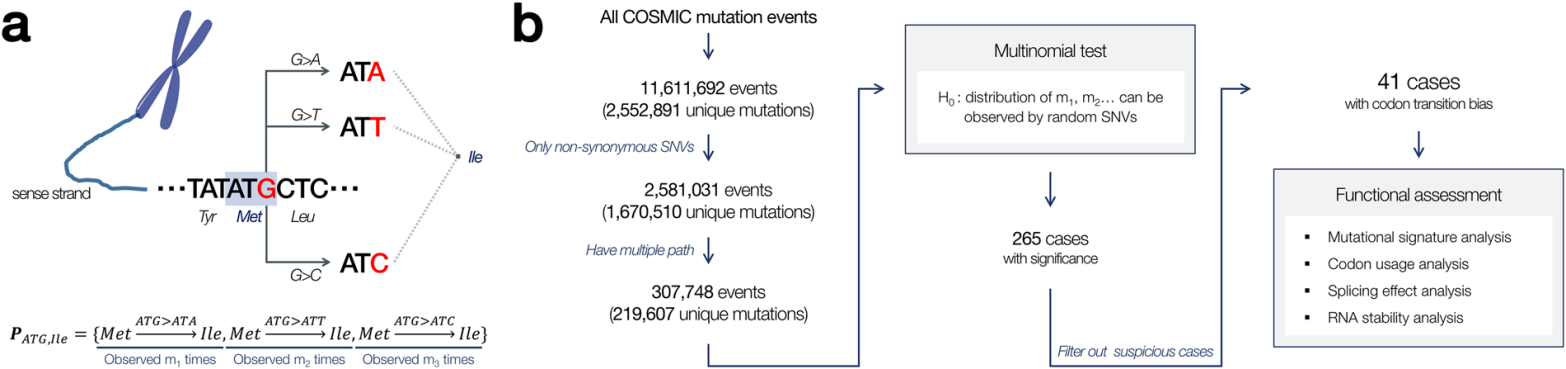
The basic concept and overall workflow. (**a**) An example of a pathset. Codon ATG in a sense strand encodes methionine, in which three mutations (G > A, G > T, G > C) lead to the alteration to isoleucine. (**b**) All somatic mutation events reported in the COSMIC database were tested for the possible codon transition bias (CTB) in a pathset. Amino acid alterations with a CTB were further analyzed for potential functionality.

Here, we extend previously known CUB to explore the above problem. Instead of measuring the usage of all codons, we interrogated whether there is any statistical deviation among synonym codon changes that encode the same amino acid alteration, defined as a synonym codon transition bias (CTB). One representative example is the methionine-to-isoleucine alteration (Fig. 1a), which is encoded by three different nonsynonymous somatic point mutations in the sense strand: Met^ATG^ to Ile^ATT^ (G > T), Ile^ATC^ (G > C), and Ile^ATA^ (G > A). A null hypothesis is that the three transitions are equally functional and happen in a balanced way due to the randomness of the mutations; practically, the nucleotide level mutation frequencies (e.g., transition-to-transversion ratio) must be considered (see Methods). On the contrary, severe deviations from the expectation may indicate the presence of additional effects at various steps within the Central Dogma. We searched for global and local (specific to a gene or a locus) CTBs in a large database of cancer somatic mutations^19^ and found 41 significantly biased transitions, most of which are located in well-known cancer-driver genes (oncogenes and tumor suppressor genes) with a sufficient number of recurrences. Multiple computational analyses were conducted to find a possible relation of the bias to post-transcriptional efficiency (e.g., RNA secondary structure, translation speed) of the genes with the mutant codons.

Overall, the extension and application of codon usage analysis to cancer somatic mutations revealed previously neglected events that can hardly occur by chance. We anticipate that the reported cancer mutations may hold clues to an unexplored mechanism of cancer cell regulation.

## Materials and Methods

### Basic definitions

We define **A** as a set of letters that denote 20 standard amino acids (e.g., “Phe,” “Leu,” and “Gly”) and one special action for translation termination (“Stop”); we regard the “Stop” letter as an amino acid for convenience of description. Nonsynonymous somatic point mutation m alters amino acids from *X* to *Y* (*X*, *Y* ∈ **A**), which we denoted as $X\mathop{\longrightarrow }\limits^{m}Y$ (e.g., $Met\mathop{\longrightarrow }\limits^{G > T}Ile$). We also define **C** as a set of 64 three-letter codons. A subset of codons **C**_*X*_ is the set of codons that encode amino acid *X*. For example, **C**_*Met*_ = {ATG} and **C**_*Ile*_ = {ATA, ATC, ATT}. Then, we define a *codon transition* with respect to the encoded amino acid as:

1 $$tr(C,C\mbox{'})=X\mathop{\longrightarrow }\limits^{C > C\mbox{'}}Y,\,{\rm{C}}\in {{\bf{C}}}_{X},\,{\rm{C}}\mbox{'}\in {{\bf{C}}}_{Y},$$

where *X* and *Y* are the amino acids encoded by C and C’, respectively. We further define a *close codon transition* as a codon transition that can be acquired via a single nucleotide variation (SNV):

2 $$tr(C,C\mbox{'})=ctr(C,C\mbox{'}),\,{\rm{i}}{\rm{f}}\,{\rm{a}}{\rm{n}}{\rm{d}}\,{\rm{o}}{\rm{n}}{\rm{l}}{\rm{y}}\,{\rm{i}}{\rm{f}}\,LD(C,C\mbox{'})=1,$$

where *LD*(C, C’) is the Levenshtein distance (or edit distance) between codons C and C’. For example, $Met\mathop{\longrightarrow }\limits^{ATG > ATT}Ile$ is a close codon transition, whereas $Met\mathop{\longrightarrow }\limits^{ATG > AGT}Ser$ is not.

Now, we define a *codon*-*amino acid transition pathset* or simply *pathset* as a set of close codon transitions from a codon C to an amino acid *Y*:

3 $${{\bf{P}}}_{{\rm{C}},{\rm{Y}}}=\{ctr(C,{C}_{i}):{C}_{i}\in {{\bf{C}}}_{Y}\},$$

where *X* is an amino acid encoded by C and *X* ≠ *Y*. By definition, a pathset is null when no SNV can convert *X* to *Y*. For other cases, the size of a pathset indicates the number of unique SNVs that result in the same amino acid alteration. For example, *N*(**P**_GTT,Ala_) = 1, where the only close codon transition is *tr*(GTT, GCT). Similarly, *N*(**P**_AGA,Ser_) = 2, because the pathset has two different close codon transitions: *tr*(AGA, AGT) and *tr*(AGA, AGC). In the rest of this study, the main focus is on pathsets of size ≥2. Intuitively, the cases show that a gene and its genomic coordinate fix the initial reference codon, and there are multiple SNVs that lead to the same target amino acid alteration. We aimed to investigate whether there is a bias among the SNVs in terms of their observed frequencies.

### Identification of codon transition bias

Let **P**_C,Y_ be a pathset with *n* close codon transitions {*ctr*(C, C_*i*_): 1 ≤ *i* ≤ *n*}. In a sufficiently large database, we assume that a total of m_i_ events of somatic nonsynonymous SNVs for *tr*(C, C_i_) are observed. Then:

4 $${\rm{m}}=\sum _{i}^{n}{m}_{i}=\sum _{i}^{n}N\,(\{ctr(C,{C}_{i}):{C}_{i}\in {{\bf{C}}}_{Y}\})=N({{\bf{P}}}_{{\rm{C}},{\rm{Y}}}),$$

where m is the total number of somatic mutations. We want to test whether there is statistical significance of deviation from a theoretically expected distribution of the observations into *n* categories. Hence, a multinomial test is applied. For a multinomial test, a vector of the observed numbers of codon transitions **m** = (*m*_*1*_, *m*_*2*_, …, *m*_*n*_) is defined with matching parameter values under the null hypothesis:

5 $${H}_{0}:{\boldsymbol{\pi }}=({{\rm{\pi }}}_{1},\,{{\rm{\pi }}}_{2},\,\ldots ,\,{{\rm{\pi }}}_{{\rm{n}}}),$$

where *π*_*i*_ is the prior probability of observing *ctr*(*C*, *C*_*i*_) out of all the possible close codon transitions in a pathset, and ${\sum }_{i}^{n}{\pi }_{i}=1$ (Fig. 1a). The naïve assignment of *π*_*i*_ can be $1/n$. Nevertheless, we note that the DNA mutation rate is specific to a sequence context: a higher mutation rate of transition (Ti: interchanges within purines or within pyrimidines) than transversion (Tv: interchanges between a purine and pyrimidine). Regarding the previously reported Ti/Tv ratios 2.0–2.1^20^, we assigned 67.75 and 32.25 to *π*_*i*_ which correspond to Ti and Tv, respectively, and further normalized them so that ${\sum }_{i}^{n}{\pi }_{i}=1$. The exact probability of the observed m under the null hypothesis is given by

6 $${\rm{\Pr }}{({\bf{m}})}_{0}={\rm{m}}!\,\prod _{i=1}^{n}\frac{{\pi }_{i}^{{m}_{i}}}{{m}_{i}!}$$

and the significance probability for the test is calculated as

7 $${\rm{\Pr }}({\bf{sig}})=\sum _{y:{\rm{\Pr }}(y)\le {\rm{\Pr }}{({\bf{m}})}_{0}}{\rm{\Pr }}(y)$$

P-values were calculated using the “dmultinom” function in R, and next they were corrected by the Bonferroni correction method. Pathsets whose corrected p-values are under 0.05 were assumed to have a synonym CTB.

### Analysis of somatic mutations in human cancer

The overall workflow is shown in Fig. 1b. In total, 11,611,692 somatic mutation events (2,552,891 unique mutations) were downloaded from the COSMIC database, version 75 (2015-NOV-24). Among them, 2,581,031 events (1,670,510 unique mutations) were nonsynonymous point mutations. Mutation events that belong to the same codon transition (codon C to amino acid *Y* in a gene) were grouped and were assigned to the same pathset **P**_C,Y_. The reading frame was determined by the annotated transcript information in the COSMIC database (“Accession Number”). When two or more events in the same gene are annotated with different transcripts, the reading frame was calculated using the Ensembl database to determine whether both are located in the same codon. In total, 307,748 mutation events (219,607 unique mutations) were mapped to codon transitions that belong to pathsets of size ≥2.

Statistical tests (see Methods above) identified 265 pathsets from 32 genes with a significant CTB. The 265 cases were further inspected for possible artefacts in mutation calling. The most important artefact is the erroneous somatic mutation call in the original studies that contributed to the COSMIC database. First, we filtered out 18 cases that were disqualified by a reference version error. We next searched for false somatic mutations. We noted that germline variants are frequently mislabeled as somatic mutations, where sequencing quality in the control sample (e.g., matching blood, saliva, or tissue near a tumor) is compromised; in this case, the germline variants look like tumor-only (somatic) mutations. Moreover, such false somatic mutations are likely to have a CTB because the source of the mutation is actually common alternative alleles (SNPs). To identify these cases, we added two filtering criteria: i) the genomic position of the pathsets is not reported to be an SNP site (no dbSNP ID) and ii) if it is a known SNP site, then the allele frequency in the population is less than 0.01. By applying these criteria, we filtered out 206 additional cases and were left with only 41 remaining high-confidence cases of a CTB.

### Functional assessment of biased codon transitions

We attempted to associate cellular functions with the identified pathsets with a CTB. The expected functions were subdivided into two major classes. One is the external factor that affects the mutational process itself. We used mutational signature analysis^21^ to identify the causes of mutations, which convert nucleotides more frequently in specific sequence contexts (e.g., tobacco smoking is associated with C > A single and CC > AA dinucleotide mutations). The other explanation of the CTB is possible alteration of transcription and translation efficiency. Therefore, we calculated three known measures for each pathset (global codon usage, a splicing effect, and mRNA stability), which we used to test whether there is a major discrepancy among the synonym codon transitions.

#### Mutational signature analysis

For this analysis, we extracted 3-bp sequences around the mutation locus (1 bp upstream and 1 bp downstream). If possible, the extracted sequence was matched to one of the previously reported 30 types of mutational signatures^22^. We then investigated the organ-specific occurrence of the mutations to confirm the assigned mutational signature. For example, if a major codon transition corresponds to the tobacco smoking signature and is specifically observed in lung cancer, CTB is well explained by the presence of a mutagen.

#### Functional effect analysis

For the other pathsets, which are not sufficiently explained by the mutational signature, functional assessment was conducted. First, we tested whether the codons that are produced by the major transition are more frequently used globally. To test the relative codon usage preference among the codons that encode a same amino acid, we used the relative synonymous codon usage (RSCU) defined by Sharp *et al*.^11^. Briefly, RSCU is calculated by global codon frequency of a specific codon divided by arithmetic mean of global codon frequency of synonym codons. The global codon frequency in a human reference genome was downloaded from the Codon Usage Database (http://www.kazusa.or.jp/codon/). Using the RSCU values, a difference in RSCU between the major and the minor codon (∆RSCU) is defined as:

8 $${\rm{\Delta }}{\rm{R}}{\rm{S}}{\rm{C}}{\rm{U}}={R}_{1}-{R}_{2},$$

where *R*_n_ is the RSCU of a codon that is the n*th* most frequently observed in a pathset. For example, two close codon transitions *tr*(*AGA*, *AGT*) and *tr*(*AGA*, *AGC*) in the previously discussed pathset **P**_AGA,Ser_ were observed 10 and 20 times respectively. By definition, *R*_1_ is the RSCU of AGC (=1.44, see Supplementary Table S1), *R*_2_ is the RSCU of AGT (=0.90), and the ∆RSCU is 0.54 (=1.44–0.90). Positive ∆RSCU implies that the favored codon transition generates a more globally preferred codon.

Second, we predicted the effect of somatic mutations on alternative splicing to test whether a major codon transition is associated with a splice aberration event. For each mutation in a given pathset, MutPred Splice version 1.3.2 with default options was used to calculate a probabilistic score for a splicing aberration and was then classified into splicing altering variants (SAVs) or splice neutral variants^23^. The predicted score was revalidated by means of another tool: Human Splicing Finder, version 3.0.0 with default options^24^.

Finally, mRNA stability was analyzed for each transcript produced by the set of mutations in a pathset. We used RNAfold version 2.2.4 to predict mRNA secondary structure^25^. We considered two types of features in the mRNA stability analysis: the free energy level of the predicted mRNA and the structural similarity with the wild-type mRNA. Among all the possible mRNA secondary structures with respect to a given mRNA sequence, the one with the lowest free energy (minimum free energy, MFE) and another with maximum accuracy (maximum expected accuracy, MEA) were used to assign free energy. The structural similarity was calculated by SimTree^26^, which outputs a normalized score between 0 to 1 (1 means that the two structures are identical). The score was further transformed to percentage similarity by multiplying by 100. Consequently, a mutation—that forms an altered mRNA transcript whose secondary structure has a lower free energy and is more similar to the structure of wild-type mRNA—is considered more stable.

## Results

### Synonym codon transitions in human cancer

**Figure 2:**
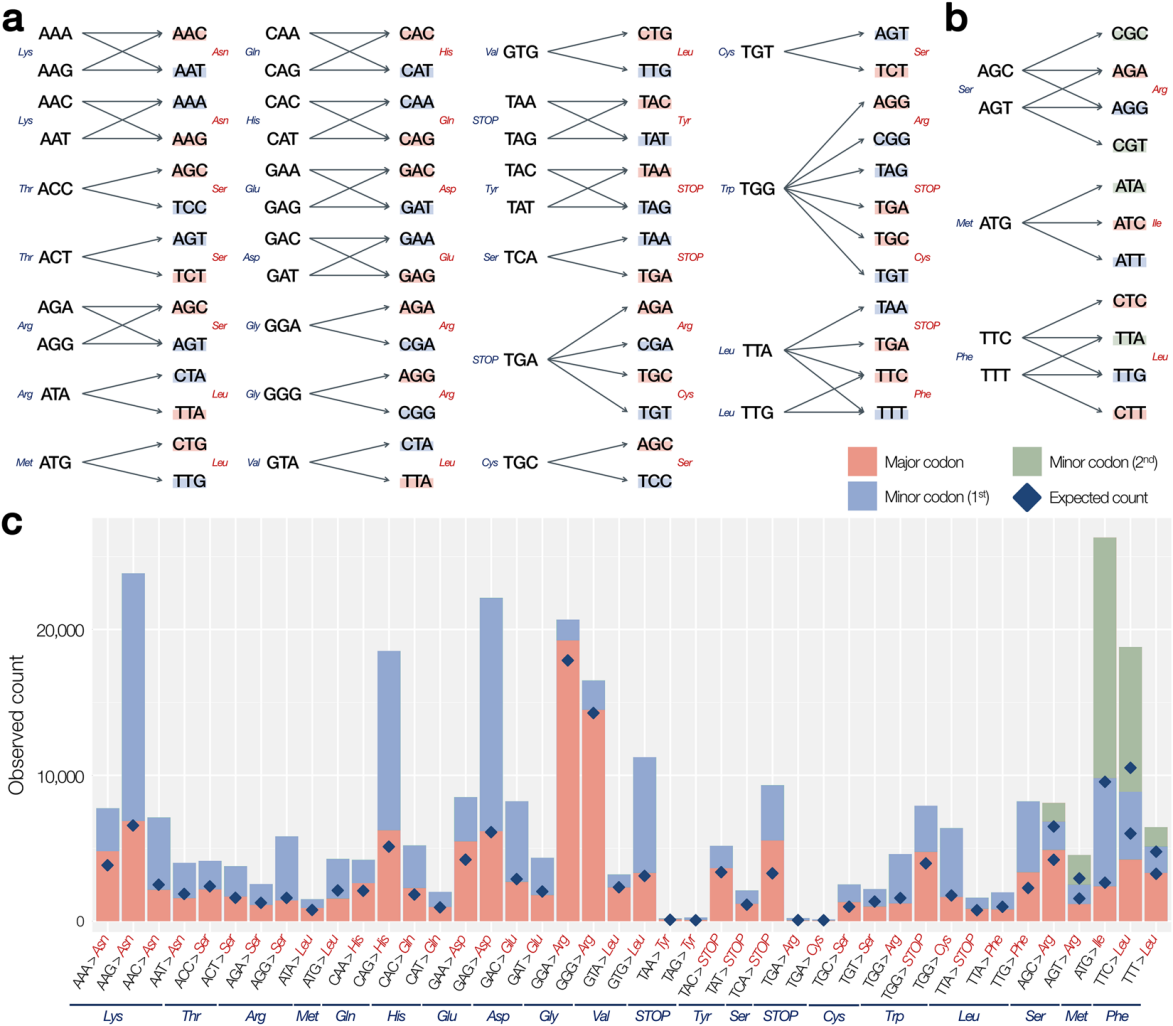
The complete list of all possible codon transitions with multiple paths and their distribution in COSMIC. (**a**) Thirty-seven pathsets with two synonym codon transitions. Colors in the target codon denote the reported general usage of the codons. (red: codons with high usage, blue: codons with low usage, green: codons with the lowest usage). (**b**) Five pathsets with three synonym codon transitions. (**c**) Distribution of codon transitions of the 42 pathsets based on the 307,748 somatic mutation events in COSMIC. The expected number of events for each synonym codon transition is indicated by the blue diamonds.

We first enumerated the possible mutation-driven codon transitions, in which two or more different nucleotide changes lead to the same amino acid alternation (*pathsets*, see Methods for a formal definition). We found 42 pathsets, 37 of which are pathsets of size 2 (Fig. 2a), and five are of size 3 (Fig. 2b). The 42 pathsets consist of 25 unique amino acid alterations, out of all possible 420 cases (_21_ ***P***_2_, the number of ordered pairs from 20 amino acids plus 1 stop codon). Therefore, formation of a pathset is a relatively rare event (5.95%). We found that most of the codon transitions are caused by the mutation at the third position of the codon. Accordingly, the codon transitions in a pathset tend to have mutations at the same codon position.

We next analyzed how the reported somatic mutation events are distributed among the 42 pathsets (Fig. 2c). In total, 307,748 somatic mutation events (219,607 unique somatic mutations) were mapped to the 42 pathsets (see Methods). The most frequently observed pathset was **P**_*ATG*,*Ile*_, which is encoded by three codon transitions: *tr*(*ATG*, *ATA*), *tr*(*ATG*, *ATT*), and *tr*(*ATG*, *ATC*) (as shown in Fig. 1a). On the contrary, pathsets that alter the stop codon (**P**_*TAA*,*Tyr*_, **P**_*TAG*,*Tyr*_, **P**_*TGA*,*Arg*_, and **P**_*TGA*,*Cys*_) were rarely observed, probably due to the higher functional impact. Nonetheless, stop causing events (**P**_*TAC*,*STOP*_, **P**_*TAT*,*STOP*_, **P**_*TCA*,*STOP*_, **P**_*TGG*,*STOP*_, and **P**_*TTA*,*STOP*_) were much more frequently observed than stop loss events were. Although there is no notable association between the number of observed events and the amino acid or codon characteristics, it is expected that a greater number of somatic mutations occur in codons with high usage.

The number of observed synonym codon transitions in the 42 pathsets shows that the codon transition is naturally biased by the sequence context. For example, pathset **P**_*GGG*,*Arg*_ consists of two synonym codon transitions *tr*(*GGG*, *AGG*) and *tr*(*GGG*, *CGG*), which are encoded by mutations G > A and G > C, respectively. It is well known that the G > A mutation (transition) occurs more frequently than G > C (transversion), leading to biased observation in the COSMIC database (Fig. 2c). Because the bias from the discrepancy between transition and transversion is not our primary concern, the expected number of observations was recalibrated by the transition/transversion ratio (Ti/Tv ratio, see Methods). We found that the corrected expected numbers (blue diamonds in Fig. 2c) successfully explain the unbalanced occurrence among synonym codon transitions.

### Identification of codon transition bias

**Figure 3:**
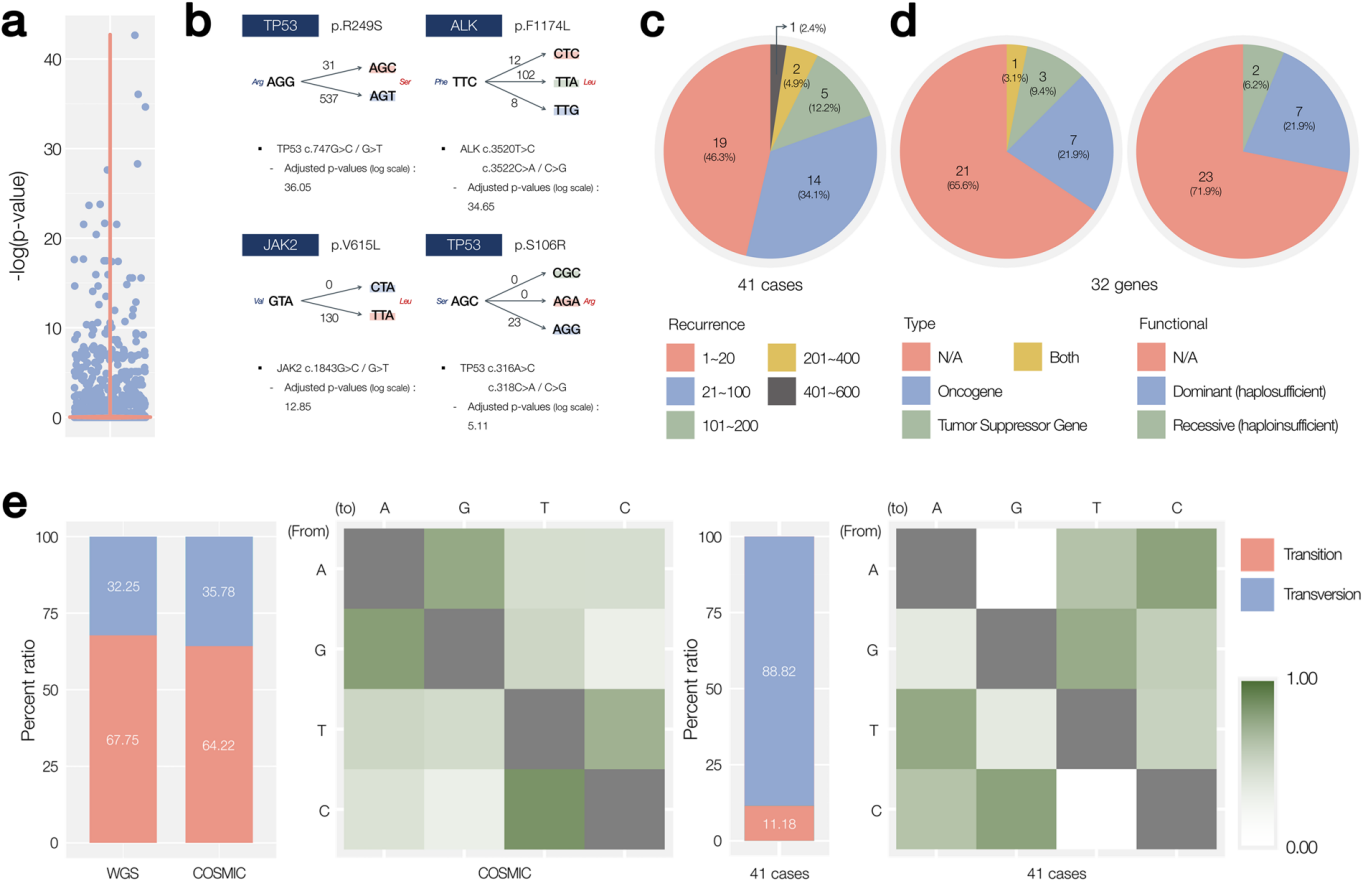
Characteristics of the 41 amino acid alterations with a CTB. (**a**) Distribution of the adjusted p-values. (**b**) Four examples of cases with a significant CTB. (**c**) The number of recurrences of the 41 alterations with a CTB. (**d**) Functional annotation of 32 genes with a CTB. The numbers of oncogenes and tumor suppressor genes are indicated with their haplosufficiency. (**e**) Comparison of the Ti/Tv ratios and mutation rates between the whole COSMIC database and 41 mutations with a CTB.

On the basis of the Ti/Tv ratio and adjusted multinomial test (see Methods), 265 amino acid alteration events were found to have a significant CTB (see Supplementary Table S2 for the entire list). The overall distribution of p-values shows that most of the amino acid alterations with possible multiple paths have no CTB (Fig. 3a), again confirming the rarity (0.12%). Out of the 265 alterations, 224 were filtered out based on our false mutation filtering criteria (see Methods), which distinguished true somatic mutations from germ line mutations using large polymorphism databases^27^. Finally, 41 amino acid alterations were finalized to have CTB (Table 1)

**Table 1:** Forty-one mutations with a CTB (arranged by p-values in ascending order).

Gene	Type	Func.	Mut AA	Mut CDS	Recur.	Impct	Gene	Type	Func.	Mut AA	Mut CDS	Recur.	Impct
TP53	TSG	Rec	R249S	AGG > AGT	537	NEU	IFITM3	—	—	G133R	GGA > CGA	11	NEU
				AGG > AGC	31	NEU					GGA > AGA	0	NEU
PIK3CA	OCG	Dom	N345K	AAT > AAA	123	PAT	TP53	TSG	Rec	F134L	TTT > CTT	41	NEU
				AAT > AAG	0	PAT					TTT > TTA	1	NEU
ALK	OCG	Dom	F1174L	TTC > TTA	102	PAT					TTT > TTG	2	NEU
				TTC > TTG	8	PAT	KCNJ5	—	Dom	G151R	GGG > AGG	169	PAT
				TTC > CTC	12	PAT					GGG > CGG	68	PAT
JAK2	OCG	Dom	V615L	GTA > TTA	130	NEU	PCLO	—	—	S1057*	TCA > TGA	20	PAT
				GTA > CTA	0	NEU					TCA > TAA	0	PAT
KRAS	OCG	Dom	Q61H	CAA > CAC	240	PAT	RYBP	—	—	G291R	GGG > CGG	10	NEU
				CAA > CAT	95	PAT					GGG > AGG	0	NEU
SIRPA	—	—	D95E	GAC > GAG	30	NEU	FGFR2	OCG	Dom	W290C	TGG > TGC	15	PAT
				GAC > GAA	0	NEU					TGG > TGT	0	PAT
SLC25A5	—	—	K296N	AAG > AAC	23	PAT	ESYT1	—	—	S214R	AGC > AGG	18	NEU
				AAG > AAT	0	PAT					AGC > AGA	0	NEU
MUC4	OCG	—	H1309Q	CAC > CAG	28	NEU					AGC > CGC	0	NEU
				CAC > CAA	0	NEU	KMT2C	TSG	Rec	T316S	ACC > TGC	25	NEU
TP53	TSG	Rec	S183*	TCA > TGA	51	NEU					ACC > AGT	1	NEU
				TCA > TAA	13	NEU	MLL3	—	—	T316S	ACC > TGC	25	NEU
LHX1	OCG	—	K204N	AAG > AAC	22	NEU					ACC > AGT	1	NEU
				AAG > AAT	0	NEU	BAT2D1	—	—	M267I	ATG > ATT	14	NEU
AGAP8	—	—	Q567H	CAG > CAC	22	NEU					ATG > ATC	0	NEU
				CAG > CAT	0	NEU					ATG > ATA	0	NEU
ZNF429	—	—	K556N	AAG > AAC	24	NEU	STRA8	—	—	E212D	GAG > GAC	14	NEU
				AAG > AAT	1	NEU					GAG > GAT	0	NEU
JPH3	—	—	V581L	GTG > CTG	21	NEU	APOBR	—	—	E352D	GAG > GAC	14	NEU
				GTG > TTG	0	NEU					GAG > GAT	0	NEU
NBPF10	—	—	K31N	AAG > AAC	19	NEU	TP53	TSG	Rec	E258D	GAA > GAT	36	PAT
				AAG > AAT	0	NEU					GAA > GAC	3	PAT
TP53	TSG	Rec	S106R	AGC > AGG	23	NEU	ADAD2	—	—	K16N	AAG > AAC	13	NEU
				AGC > AGA	0	NEU					AAG > AAT	0	NEU
				AGC > CGC	0	NEU	BNC2	—	—	S575R	AGT > CGT	16	NEU
PIK3CA	OCG	Dom	M1043I	ATG > ATT	57	PAT					AGT > AGA	0	NEU
				ATG > ATA	38	PAT					AGT > AGG	0	NEU
				ATG > ATC	10	PAT	GIGYF2	—	—	G108R	GGA > CGA	8	PAT
TP53	TSG	Rec	K132N	AAG > AAC	82	PAT					GGA > AGA	0	PAT
				AAG > AAT	76	PAT	NPM1	Both	Dom	G90R	GGG > CGG	8	PAT
FAM75A3	—	—	E950D	GAG > GAC	18	NEU					GGG > AGG	0	PAT
				GAG > GAT	0	NEU	TP53	TSG	Rec	S215R	AGT > AGG	33	NEU
CCDC136	TSG	—	Q396H	CAG > CAC	18	NEU					AGT > CGT	7	NEU
				CAG > CAT	0	NEU					AGT > AGA	19	NEU
SLC25A5	—	—	F271L	TTC > CTC	20	NEU	APOBR	—	—	E361D	GAG > GAC	12	NEU
				TTC > TTA	0	NEU					GAG > GAT	0	NEU
				TTC > TTG	0	NEU	ASPM	—	—	K3446N	AAG > AAC	12	NEU
POU2F2	—	—	S449R	AGC > CGC	14	NEU					AAG > AAT	0	NEU
				AGC > AGA	0	NEU							
				AGC > AGG	0	NEU							

*Abbreviations: Func.: Haplosufficiency. Recur.: Recurrence. Impct: FATHMM prediction result. Mut AA: Amino acid mutation. Mut CDS: Codon alteration. OCG: Oncogene. TSG: Tumor Suppressor Gene. Both: OCG & TSG. Dom: Dominant. Rec: Recessive. NEU: Neutral. PAT: Pathogenic.

A few representative examples are shown in Fig. 3b. The *TP53* p.R249S is a well-known somatic amino acid alteration in a tumor suppressor, which has been reported 568 times in the COSMIC database. *TP53* p.R249S has two synonym codon transitions, *tr*(*AGG*, *AGC*) and *tr*(*AGG*, *AGT*), which are encoded by mutations G > C and G > T, respectively, at the third position of the codon. Moreover, we found that the G > T mutations occurred much more frequently than G > C (537 times vs. 31 times, corrected p-value < 10^−36^). The two mutations are both transversions, which does not explain the difference sufficiently. *ALK* p.F1174L is another frequently reported somatic amino acid alteration in an oncogene. Pathset **P**_*TTC*,*Leu*_ has three synonym codon transitions: *tr*(*TTC*, *CTC*), *tr*(*TTC*, *TTA*), and *tr*(*TTC*, *TTG*). The most frequent codon transition was *tr*(*TTC*, *TTA*), which was caused by transversion C > A at the third position of the codon (108 times vs. 12 and 8 times, corrected p-value < 10^−34^). Likewise, two other examples in *JAK2* and another *TP53* are shown in Fig. 3b.

We conducted functional classification of the 41 amino acid alterations with a CTB (Fig. 3c,d). Frequent recurrence of a mutation in cancer is an important factor determining the pathogenicity of cancer mutation. The number of recurrences was at least 8 (2 out 41 cases). In more than a half of all cases (22/41, ~54%), the same alterations were observed more than 20 times. There were eight cases with >100 occurrences, convincing a functional relation with cancer (Fig. 3c). The 41 alterations occurred in 32 unique genes, seven and three of which are classified as oncogenes and tumor suppressor genes, respectively; the classification was based on the databases by Min Zhao *et al*.^28,29^. The high prevalence of oncogenes and tumor suppressor genes implies a potential functional association with cancer initiation and progression. Because most of oncogenes are pathogenic because of one mutant allele, a similar distribution was observed in the haplosufficiency analysis (Fig. 3d).

The analysis of the Ti/Tv ratio also supports functionality of the 41 alterations. Under normal conditions, the Ti/Tv ratio is between 2.1 and 2.7 as observed in normal whole genome sequencing and the COSMIC database (Fig. 3e, left bar graph). Accordingly, the heatmap of nucleotide changes shows enriched transition mutations (Fig. 3e, the second heatmap). On the contrary, the Ti/Tv ratio of the 41 alterations was only 0.13 (11.18/88.82), and the corresponding heatmap shows an asymmetrical pattern of preferred nucleotide changes (Fig. 3e, right). Therefore, the somatic mutations within the selected 41 cases with a CTB are assumed to have a strong functional association with cancer.

### Analysis of potential causes and effects of codon transition bias

The molecular basis of an extreme CTB is unknown and requires further experiments for elucidation and confirmation. Nonetheless, several *in silico* analyses provide a partial explanation for the cause of a CTB and its potential effects.

**Figure 4:**
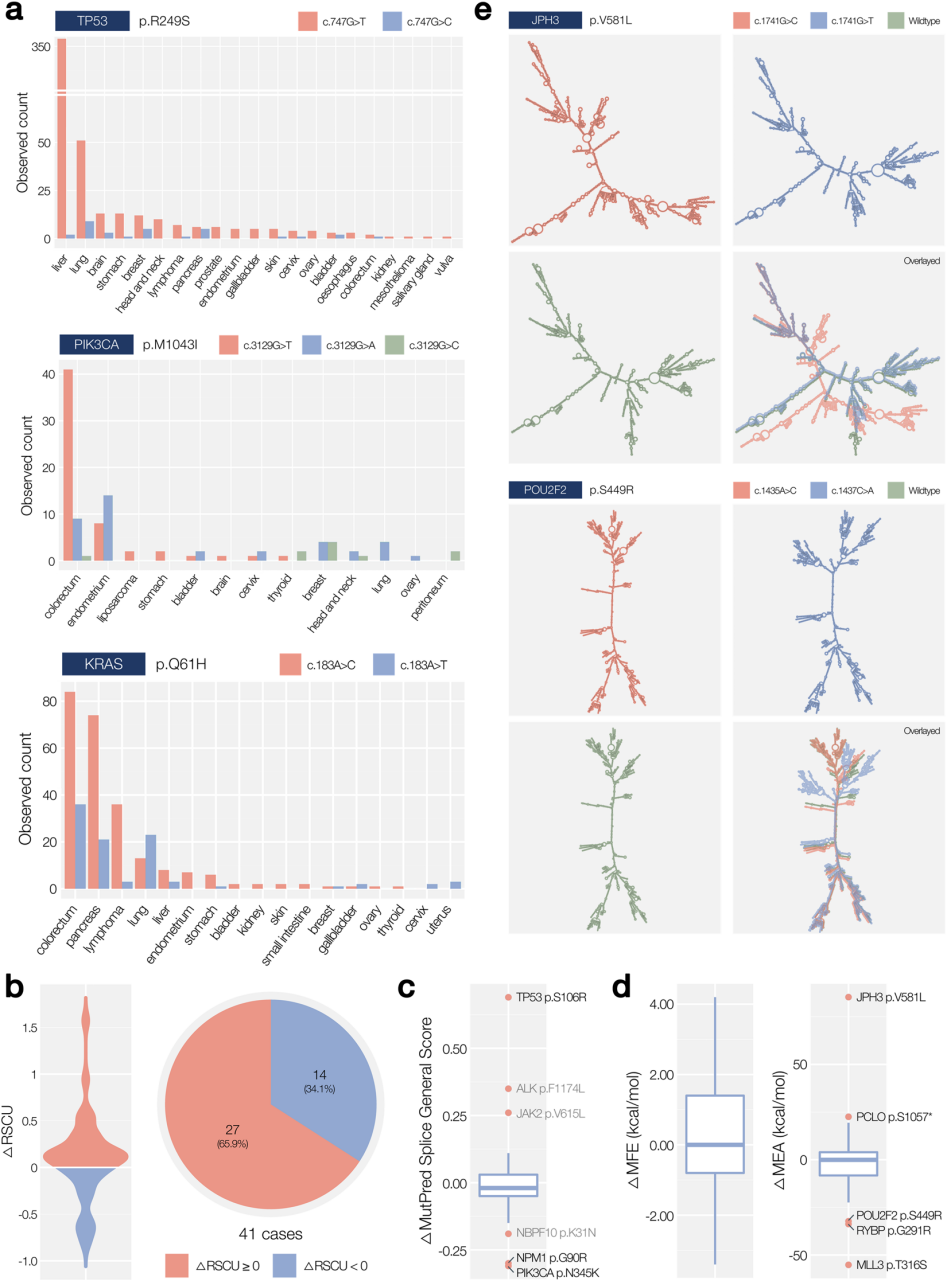
Functional assessment results. (**a**) Distribution by histology and tissues of origin in cases related to mutational signatures and tissue specificity. (**b**) Distribution of the gap between codon usage rates (left) and the quantitative status (right). (**c**) Distribution of predicted effect sizes on splicing. (**d**) Distribution of differences in the MFE (left) and MEA (right) of the codons in the pathset associated with RNA stability. (**e**) Two extreme cases of maximum expected accuracy of free energy of a conformation’s secondary structure (*JPH3* p.V581L and *POU2F2* p.S449R).

First, we conducted mutational signature analysis combined with tissue specificity to determine whether a specific mutational process is involved in the favored codon transition. The *TP53* p.R249S is one exemplary case, in which the mutation signature was useful. We found that the overwhelming occurrence of *tr*(*AGG*, *AGT*) (mutation G > T, observed 537 times) over *tr*(*AGG*, *AGC*) (mutation G > C, observed 31 times) was intensified in the liver and lungs (Fig. 4a, top). Moreover, a recent population-specific study revealed that hepatocellular carcinoma that develops in the absence of liver cirrhosis frequently harbors the *TP53* p.R249S mutation caused by aflatoxin B1^30^. Aflatoxin B1 is a top-tier carcinogen in the liver and lungs that produces the G > T mutation induced by 8-hydroxy-2′-deoxyguanosine^31^. Thus, the extreme CTB in *TP53* p.R249S can be caused by the aflatoxin B1-induced liver and lung cancer. Another example is *PIK3CA* p.M1043I, which consists of three synonym codon transitions: *tr*(*ATG*, *ATT*), *tr*(*ATG*, *ATA*), and *tr*(*ATG*, *ATC*). The G > T mutation (c.3129G>T) is the most frequent codon transition (observed 57 times vs. 38 and 10 times for G > A and G > C, respectively; Fig. 4a, middle). Nevertheless, we found that the dominance of the G > T mutation was observed only in colorectal cancer, whereas the G > A mutation was more frequent in other tissues. One of the known mutational signatures, mismatch repair deficiency, is represented by C > T (G > A) and C > A (G > T) mutations in cancers with microsatellite instability (MSI)^32^. It is well known that almost 15% of colorectal cancers have the MSI signature. The G > A mutation is also known to be related to aggressive endometrial cancer^33,34^. A similar pattern was discovered in *KRAS* p.Q61H, whose major codon transition c.183A>C is observed in colorectal and pancreatic cancers (Fig. 4a, bottom)

Next, we tested whether the codon transition is biased with usage. Out of the 41 amino acid alterations with a CTB, 27 cases (65.9%) were biased toward generation of a high usage codon (Fig. 4b, see Supplementary Table S3 for the ∆RSCU of them). The distribution of the gap between the codon usage rates of the favored and unflavored codons (∆RSCU, see Methods) identified a few cases with large deviations. Although codon usage does not resolve the entire case, the tendency toward higher usage suggests that mRNAs with a more efficient conformation (e.g., faster translation elongation) would be favored by cancer cells. Particularly, recent studies that reported alteration of tRNA expression in tumor cell can provide a basis for interpretation of our findings in terms of increased translational efficiency^10^.

We then determined whether some codon transitions are related to the *de novo* formation or loss of mRNA splicing. As shown by recent studies, mRNA splicing is not only determined by the canonical splicing donor and acceptor but also affected by various motifs in introns or exons that regulate activity of the spliceosome: spice enhancers and silencers^35^. We annotated all the somatic mutations involved in the 41 alterations with the predicted effect size on splicing (see Fig. 4c and Methods). The predicted scores converged on approximately zero, which means that splicing was not additionally disrupted in most of the codon transitions. Nonetheless, we found six cases whose scores strongly deviated from the average including *TP53* p.S106R and *PIK3CA* p.N345K (Table 2). Out of the three possible synonym codon transitions in *TP53* p.S106R, only the favored mutation C > G was observed (23 times vs. 0 times). The C > G mutations are predicted to generate a new donor site as well as a new exonic splice enhancer. The disruption in the structural regulation of *TP53* may accelerate the breakdown of its function as a tumor suppressor and can be favored by a cancer cell. In contrast, splicing affecting codon transition *tr*(*AAT*, *AAA*) is avoided in *PIK3CA* p.N345K (0 vs. 123 times, Table 2). We believe that the *de novo* generation of a splice acceptor may lead to disruption of the oncogene itself, thereby preventing proliferation of the cells. Although the exact mechanisms should be validated in further studies, the different effects on mRNA splicing and their mode of action must be considered with respect to the original function of the genes.

**Table 2:** Mutation with a CTB predicted to have a splicing effect.

Gene	Type	HaploSufficiency	Amino acid Mutation	CodonAlteration	Recur.	MSGS	HSF Predicted Signal		
							Splice site	ESS	ESE
TP53	TSG	Rec	S106R	AGC > AGG	23	0.97	O(ND)	O	O
				AGC > AGA	0	0.28			O
				AGC > CGC	0	0.22			
NPM1	Both	Dom	G90R	GGG > CGG	8	0.41			
				GGG > AGG	0	0.71	O(NA)	O	
PIK3CA	OCG	Dom	N345K	AAT > AAA	123	0.58			
				AAT > AAG	0	0.89	O(NA)	O	O
KCNJ5	—	Dom	G151R	GGG > AGG	169	0.81			
				GGG > CGG	68	0.70			
ESYT1	—	—	S214R	AGC > AGG	18	0.93		O	
				AGC > AGA	0	0.82		O	O
				AGC > CGC	0	N/A	O(BD)		O

*Abbreviations: Recur.: Recurrence. MSGS: MutPred Splice General Score. HSF: Human Splice Finder. OCG: Oncogene. TSG: Tumor Suppressor Gene. Both: OCG & TSG. ESS: New Exonic Splicing. Silencers (ESS) site. ESE: Exonic Splicing Enhancers (ESE) site broken. NA: New Acceptor site. ND: New Donor site. BD: Broken WT Donor site. Dom: Dominant. Rec: Recessive.

Finally, we analyzed the effects of synonym codon transitions on mRNA stability (Fig. 4d). To measure the stability, secondary structures of mRNAs were predicted using two options (MFE and maximum expected accuracy: MEA, see Methods). For each predicted conformation, free energy was calculated. We first tested whether the free energy of the favored mRNA conformation strongly deviates from that of the avoided conformation. We found that the gap between the free energies is negligible in most cases (~0 kcal/mol), with only a few outliers including *JPH3* p.V581L and *POU2F2* p.S449R (see Fig. 4d). In addition to the different free energies, the synonym codon transitions generated mRNAs of highly different secondary structures (Fig. 4e). The frequently observed structure of *JPH3* p.V581L (c.1741G>C, 21 times vs. 0 times) was predicted to be less stable (higher free energy). In addition, the structural similarity with the wild-type *JPH3* mRNA was much lower as compared to the avoided conformation (69.79% vs. 95.38%). In another case of *POU2F2* p.S449R, the favored conformation is less stable than the other one, but the similarity to the wild-type structure was much higher (94.50% vs. 79.04%), showing that the increased mRNA stability is not always favored among the synonym codon transitions.

## Discussion

In all the statistical analyses and further *in silico* analyses, our study has some limitations. First, the biased observed frequency among different codon transitions can be caused by many factors that we did not consider. A mutational signature is only a part of clustering methods for identifying somatic mutation patterns. Any distant functional elements can affect DNA mutations at a specific site, and this effect can even be transient. There are many environmental or other external factors (e.g., carcinogen or virus) that cause a specific type of mutation, thereby leading to a biologically irrelevant codon transition bias. For example, tobacco smoking, aflatoxin and HPV infection can cause a genuine signature. Due to the limited sample and clinical information in the COSMIC database, we could not subgroup patients with different clinical characteristics. Further collection of somatic mutations with rich clinical annotation will enable a more accurate analysis of those factors. Second, the computational predictions used in this study are relatively less robust due to the stochastic nature of mRNA splicing and structure. Thus, the functional mechanisms of a CTB in a specific gene should be confirmed in a deeper experimental study. Sufficient expression data of protein or RNAs in cancer gene would help to validate it. Third, integrity of the COSMIC database can compromise the reliability of our entire study. As we already found at the filtering step (see Methods), the COSMIC database contains many false somatic mutations that are mislabeled by mutation callers. The continuous updates of the database should lead to a more reliable analysis on a larger scale in the future.

## Conclusions

In this study, multiple sets of mutations that lead to the same amino acid substitution were grouped and analyzed for a bias in a large-scale database of cancer somatic mutations. We identified 41 recurrent and potentially cancer-associated amino acid alterations with a significant CTB. The cause and possible effects of the CTB were also studied with respect to a mutational signature, codon usage, splicing effects, and mRNA stability. This is the first study to analyze cancer somatic mutations at the codon level, and this approach may uncover previously unexplored mechanisms that regulate cancer initiation and maintenance.

## Electronic supplementary material

Supplementary Info
