# Supplementary material for: Somatic mutation driven codon transition bias in human cancer
Source: Sci Rep. 2017 Oct 27;7:14204. doi: 10.1038/s41598-017-14543-1 (PMC5660177; doi:10.1038/s41598-017-14543-1)
Supplement: Supplementary file 1 — Supplementary Info [file 41598_2017_14543_MOESM1_ESM.pdf]

Somatic mutation driven codon transition bias in human cancer

Hyeonju Son[1]**, Hyundeok Kang**[1]**, Hyun Seok Kim**[1] **and Sangwoo Kim**[1,*]

[1] Severance Biomedical Science Institute, Brain Korea 21 PLUS Project for Medical Sciences, Yonsei University College of Medicine, Seoul 03722, South Korea
[*] To whom correspondence should be addressed. Tel: +82-2-2228-0913; Fax: +82-2-2227-8129; Email: swkim@yuhs.ac

Supplementary Table S1. Relative synonymous codon usage table of *Homo sapiens*

UUU	(Phe, F)	0.93	UCU	(Ser, S)	1.12	UAU	(Tyr, Y)	0.89	UGU	(Cys, C)	0.91
UUC	(Phe, F)	1.07	UCC	(Ser, S)	1.31	UAC	(Tyr, Y)	1.11	UGC	(Cys, C)	1.09
UUA	(Leu, L)	0.46	UCA	(Ser, S)	0.90	UAA	(Stop, *)	0.88	UGA	(Stop, *)	1.41
UUG	(Leu, L)	0.78	UCG	(Ser, S)	0.33	UAG	(Stop, *)	0.71	UGG	(Trp, W)	1.00
CUU	(Leu, L)	0.80	CCU	(Pro, P)	1.15	CAU	(His, H)	0.84	CGU	(Arg, R)	0.48
CUC	(Leu, L)	1.18	CCC	(Pro, P)	1.30	CAC	(His, H)	1.16	CGC	(Arg, R)	1.10
CUA	(Leu, L)	0.43	CCA	(Pro, P)	1.11	CAA	(Gln, Q)	0.53	CGA	(Arg, R)	0.66
CUG	(Leu, L)	2.35	CCG	(Pro, P)	0.45	CAG	(Gln, Q)	1.47	CGG	(Arg, R)	1.21
AUU	(Ile, I)	1.08	ACU	(Thr, T)	0.98	AAU	(Asn, N)	0.94	AGU	(Ser, S)	0.90
AUC	(Ile, I)	1.41	ACC	(Thr, T)	1.42	AAC	(Asn, N)	1.06	AGC	(Ser, S)	1.44
AUA	(Ile, I)	0.51	ACA	(Thr, T)	1.14	AAA	(Lys, K)	0.87	AGA	(Arg, R)	1.29
AUG	(Met, M)	1.00	ACG	(Thr, T)	0.46	AAG	(Lys, K)	1.13	AGG	(Arg, R)	1.27
GUU	(Val, V)	0.72	GCU	(Ala, A)	1.06	GAU	(Asp, D)	0.93	GGU	(Gly, G)	0.65
GUC	(Val, V)	0.96	GCC	(Ala, A)	1.60	GAC	(Asp, D)	1.07	GGC	(Gly, G)	1.35
GUA	(Val, V)	0.47	GCA	(Ala, A)	0.91	GAA	(Glu, E)	0.85	GGA	(Gly, G)	1.00
GUG	(Val, V)	1.85	GCG	(Ala, A)	0.43	GAG	(Glu, E)	1.15	GGG	(Gly, G)	1.00

Supplementary Table S2. Significant CTB list

Gene	Mut AA	Count	p-value	adjusted p-value
MUC6	M2064I	48	1.01E-48	2.22E-43
TP53	R249S	568	4.02E-42	8.83E-37
MUC4	M1535I	40	1.01E-40	2.22E-35
PIK3CA	N345K	123	2.32E-34	5.09E-29
PCDHA8	G97R	38	1.10E-33	2.42E-28
AK2	K181N	52	7.63E-30	1.68E-24
NBPF9	M677I	50	1.01E-29	2.22E-24
CNTNAP3B	M1247I	27	1.01E-27	2.22E-22
NCOR1	K178N	48	1.33E-27	2.92E-22
PSG4	Q11H	48	1.33E-27	2.92E-22
MUC12	V20L	46	1.75E-26	3.84E-21
OR1L6	M259I	23	1.00E-23	2.20E-18
RBMX	K9N	41	1.10E-23	2.42E-18
BCORL1	F111L	46	1.52E-23	3.34E-18
MUC6	F1585L	46	1.52E-23	3.34E-18
RGPD4	W1646R	49	1.80E-23	3.95E-18
MUC6	H1833Q	50	1.92E-23	4.22E-18
MUC4	Q3565H	38	5.27E-22	1.16E-16
OR1D5	R290S	38	5.27E-22	1.16E-16
ADAM21	D95E	46	1.26E-21	2.77E-16
RPSAP58	H36Q	46	1.26E-21	2.77E-16
AKAP8L	H458Q	46	1.26E-21	2.77E-16
OBSCN	V2080L	36	6.95E-21	1.53E-15
ZFP62	M1I	20	1.00E-20	2.20E-15
MUC4	H4205Q	44	1.02E-20	2.24E-15
ALK	F1174L	122	4.04E-20	8.87E-15
HCAR3	F198L	38	1.41E-19	3.10E-14
JAK2	V615L	130	6.43E-19	1.41E-13
MUC6	V2066L	32	1.21E-18	2.66E-13
RPSAP58	M10I	30	4.01E-18	8.81E-13
ANKRD30B	F477L	38	5.43E-18	1.19E-12
TXNRD2	S299R	24	2.08E-17	4.57E-12
KRAS	Q61H	335	9.53E-17	2.09E-11
MUC4	H3741Q	35	1.25E-16	2.75E-11
TAS2R31	F71L	25	1.29E-16	2.83E-11
LEPR	K656N	28	2.10E-16	4.61E-11
MUC4	Q2925H	28	2.10E-16	4.61E-11
CDC27	L173F	52	3.66E-16	8.04E-11
HNRNPCL1	F115L	27	4.08E-16	8.96E-11
ANKRD43	F545L	24	5.59E-16	1.23E-10
KCNJ12	M302I	26	8.37E-16	1.84E-10
ZNF469	G2358R	17	1.80E-15	3.95E-10

EFCAB2	G80R	17	1.80E-15	3.95E-10
MUC6	L1685F	48	2.23E-15	4.90E-10
MUC12	L4568F	26	2.76E-15	6.06E-10
MUC12	L4711F	26	2.76E-15	6.06E-10
ANKRD36C	L496*	44	2.88E-15	6.32E-10
STK11	F354L	32	2.89E-15	6.35E-10
MUC6	K1941N	46	9.09E-15	2.00E-09
KCNJ12	Q192H	25	1.00E-14	2.20E-09
OR4C3	V142L	25	1.00E-14	2.20E-09
KRTAP4-8	C95S	34	1.66E-14	3.65E-09
SIRPA	D95E	30	2.34E-14	5.14E-09
ZFPM1	E444D	24	3.64E-14	7.99E-09
C21orf62	F181L	23	4.95E-14	1.09E-08
IGDCC4	S1011R	19	6.22E-14	1.37E-08
HLA-A	T345S	35	8.50E-14	1.87E-08
MUC4	F1046L	44	8.51E-14	1.87E-08
SLC25A5	K296N	23	1.32E-13	2.90E-08
MPP2	V105L	23	1.32E-13	2.90E-08
TCF15	V114L	23	1.32E-13	2.90E-08
CUX2	V1472L	23	1.32E-13	2.90E-08
MPP2	V81L	23	1.32E-13	2.90E-08
MUC4	H1309Q	28	1.90E-13	4.17E-08
MUC4	H3837Q	28	1.90E-13	4.17E-08
NOTCH2NL	T196S	34	2.01E-13	4.41E-08
HLA-A	T351S	34	2.01E-13	4.41E-08
TP53	S183*	64	3.19E-13	7.01E-08
MUC6	T1807S	52	3.74E-13	8.21E-08
ANO9	F93L	25	3.96E-13	8.70E-08
EFTUD1	E478D	22	4.80E-13	1.05E-07
LHX1	K204N	22	4.80E-13	1.05E-07
MUC12	L1735F	22	4.80E-13	1.05E-07
MUC12	L1878F	22	4.80E-13	1.05E-07
HLA-DQA1	Q152H	22	4.80E-13	1.05E-07
AGAP8	Q567H	22	4.80E-13	1.05E-07
VCX2	V110L	22	4.80E-13	1.05E-07
C19orf44	D346E	27	5.41E-13	1.19E-07
PRAMEF1	F185L	27	5.41E-13	1.19E-07
LOC65121	F430L	27	5.41E-13	1.19E-07
PRAMEF1	F430L	27	5.41E-13	1.19E-07
HNRNPCL1	S286R	27	5.41E-13	1.19E-07
HOMER3	S339R	27	5.41E-13	1.19E-07
TCP10L2	E277D	40	6.17E-13	1.35E-07
ZNF429	K556N	25	6.59E-13	1.45E-07
RBMX	G379R	14	7.20E-13	1.58E-07

KRTAP2-2	W94*	40	9.09E-13	2.00E-07
MAGIX	F264L	24	1.24E-12	2.72E-07
MAGIX	F323L	24	1.24E-12	2.72E-07
PCDHA4	G72R	18	1.52E-12	3.34E-07
CACNA1B	N167K	26	1.54E-12	3.38E-07
NBPF20	N59K	26	1.54E-12	3.38E-07
DCP1B	Q252H	21	1.74E-12	3.82E-07
JPH3	V581L	21	1.74E-12	3.82E-07
TPSB2	V76L	21	1.74E-12	3.82E-07
MUC6	T1806S	48	3.38E-12	7.42E-07
DCP2	F16L	23	3.89E-12	8.54E-07
OR4M2	F225L	23	3.89E-12	8.54E-07
TLR5	F822L	23	3.89E-12	8.54E-07
MUTYH	Q335H	20	6.32E-12	1.39E-06
MUC4	Q3693H	20	6.32E-12	1.39E-06
PPIAL4G	F112L	19	1.02E-11	2.24E-06
ANKRD36C	D501E	40	1.15E-11	2.53E-06
ANKRD36	D559E	24	1.25E-11	2.75E-06
THBD	S149R	24	1.25E-11	2.75E-06
NBPF10	K31N	19	2.30E-11	5.05E-06
C20orf80	E159D	22	2.78E-11	6.11E-06
TCP10	D110E	23	3.55E-11	7.80E-06
MUC4	D3317E	23	3.55E-11	7.80E-06
MPP2	H104Q	23	3.55E-11	7.80E-06
GLYR1	H459Q	23	3.55E-11	7.80E-06
MPP2	H80Q	23	3.55E-11	7.80E-06
WDR87	N1885K	23	3.55E-11	7.80E-06
WDR87	N1924K	23	3.55E-11	7.80E-06
TP53	S106R	23	3.55E-11	7.80E-06
NAB2	F208L	21	3.82E-11	8.39E-06
PLK5	G323R	12	3.91E-11	8.59E-06
GCNT7	D230E	38	4.06E-11	8.92E-06
PIK3CA	M1043I	105	4.46E-11	9.79E-06
TP53	K132N	158	5.82E-11	1.28E-05
AQP7	E202D	18	8.33E-11	1.83E-05
FAM75A3	E950D	18	8.33E-11	1.83E-05
MUC4	Q1165H	18	8.33E-11	1.83E-05
CCDC136	Q396H	18	8.33E-11	1.83E-05
SPTBN4	V1105L	18	8.33E-11	1.83E-05
FRG2	M100I	10	1.00E-10	2.20E-05
NOTCH2NL	S181R	53	1.09E-10	2.39E-05
SLC25A5	F271L	20	1.20E-10	2.64E-05
PARD3B	N942K	52	1.69E-10	3.71E-05
POU2F2	S449R	14	1.86E-10	4.08E-05

METTL15	N31K	51	2.60E-10	5.71E-05
AGAP7	D600E	21	2.88E-10	6.32E-05
IFITM3	G133R	11	2.88E-10	6.32E-05
ADAMTSL3	G713R	11	2.88E-10	6.32E-05
FRG2B	H11Q	21	2.88E-10	6.32E-05
NPSR1	S241R	21	2.88E-10	6.32E-05
XKR3	F255L	15	2.93E-10	6.43E-05
TAS2R43	F294L	15	2.93E-10	6.43E-05
MUC4	Q4125H	17	3.03E-10	6.65E-05
TP53	F134L	44	3.72E-10	8.17E-05
GPR109B	F198L	19	3.75E-10	8.24E-05
MUC6	Y1920*	50	4.01E-10	8.81E-05
PABPC3	M251I	16	5.27E-10	0.000115733
KCNJ5	G151R	237	5.82E-10	0.000127811
CHD4	E139D	65	8.02E-10	0.000176125
PCLO	S1057*	20	8.19E-10	0.000179858
FRG2B	M100I	9	1.00E-09	0.000219607
LILRA6	M317I	9	1.00E-09	0.000219607
MUC4	M3407I	9	1.00E-09	0.000219607
HLA-C	E201D	16	1.10E-09	0.000241568
HLA-DRB1	Q178H	16	1.10E-09	0.000241568
GOLGA6L2	Q503H	16	1.10E-09	0.000241568
RP11-529J17.2	V466L	16	1.10E-09	0.000241568
HRNR	H2181Q	27	1.19E-09	0.000261332
TRIM50	W13R	48	1.70E-09	0.000373332
RYBP	G291R	10	2.12E-09	0.000465567
TMEM194B	G2R	10	2.12E-09	0.000465567
PDE4DIP	D1910E	46	2.26E-09	0.000496312
TRIML1	S254R	19	2.33E-09	0.000511684
Q8IXV1	H117Q	26	2.56E-09	0.000562194
GXYLT1	H148Q	26	2.56E-09	0.000562194
ZNF141	T325S	23	2.58E-09	0.000566586
FMR1	K119N	28	2.84E-09	0.000623684
PDE4DIP	W1396R	46	3.94E-09	0.000865252
GOLGA6L2	W191R	46	3.94E-09	0.000865252
KLRC3	W19R	46	3.94E-09	0.000865252
NBPF3	K536N	15	3.99E-09	0.000876232
NBPF3	K548N	15	3.99E-09	0.000876232
TPSAB1	V76L	15	3.99E-09	0.000876232
FGFR2	W290C	15	3.99E-09	0.000876232
BCLAF1	S718R	25	5.47E-09	0.00120125
NBPF10	F35L	18	6.64E-09	0.00145819
ESYT1	S214R	18	6.64E-09	0.00145819
KMT2C	T316S	26	6.92E-09	0.00151968

MLL3	T316S	26	6.92E-09	0.00151968
BAT2D1	M267I	14	7.61E-09	0.001671209
PCSK5	M679I	8	1.00E-08	0.00219607
PDPR	N662K	24	1.17E-08	0.002569402
OR4C3	T17S	21	1.44E-08	0.003162341
STRA8	E212D	14	1.45E-08	0.003184302
APOBR	E352D	14	1.45E-08	0.003184302
ENSG00000188219	E378D	14	1.45E-08	0.003184302
ZNF434	E92D	14	1.45E-08	0.003184302
BZRAP1	K1414N	14	1.45E-08	0.003184302
ZNF98	K560N	14	1.45E-08	0.003184302
CCBL2	K9N	14	1.45E-08	0.003184302
COIL	L66F	14	1.45E-08	0.003184302
DCP1B	Q150H	14	1.45E-08	0.003184302
MUC4	Q1757H	14	1.45E-08	0.003184302
TAS2R30	Q210H	14	1.45E-08	0.003184302
SRA1	V110L	14	1.45E-08	0.003184302
FANCI	C742S	36	1.54E-08	0.003381948
MUC6	H1513Q	48	1.70E-08	0.003733319
HLA-A	N90K	28	2.21E-08	0.004853315
TP53	E258D	39	2.28E-08	0.00500704
OR4C3	Q32H	25	2.34E-08	0.005138804
RGMA	D415E	23	2.51E-08	0.005512136
RGMA	D423E	23	2.51E-08	0.005512136
MUC6	Q1821H	54	2.78E-08	0.006105075
ANKRD36	W621R	41	3.23E-08	0.007093306
PRSS3	T124S	20	3.40E-08	0.007466638
MUC4	T1690S	20	3.40E-08	0.007466638
PRSS3	T67S	20	3.40E-08	0.007466638
PRSS3	T81S	20	3.40E-08	0.007466638
HLA-DQA1	F179L	15	3.62E-08	0.007949773
PRDM10	E444D	24	4.72E-08	0.01036545
ACVR2A	K315N	24	4.72E-08	0.01036545
NADSYN1	Q204H	24	4.72E-08	0.01036545
HLA-DPB1	E63D	13	5.26E-08	0.011551328
ADAD2	K16N	13	5.26E-08	0.011551328
FCGBP	Q2719H	13	5.26E-08	0.011551328
ATP10A	R1298S	13	5.26E-08	0.011551328
MUC12	R363S	13	5.26E-08	0.011551328
MUC12	R506S	13	5.26E-08	0.011551328
OR4N4	V117L	13	5.26E-08	0.011551328
CLIP1	L271F	52	5.29E-08	0.01161721
CRB2	H119Q	22	5.36E-08	0.011770935
ENSG00000174501	L1245*	22	5.36E-08	0.011770935

MUC4	H3325Q	16	5.38E-08	0.011814857
FAM86B2	S17*	16	5.38E-08	0.011814857
OR4C3	W174*	24	5.96E-08	0.013088577
BNC2	S575R	16	6.26E-08	0.013747398
C17orf72	T24S	30	6.77E-08	0.014867394
PLIN4	K860N	24	7.51E-08	0.016492486
MUC4	E2149D	23	9.54E-08	0.020950508
SLC6A7	M514L	23	9.54E-08	0.020950508
HLA-DQB1	Q253H	23	9.54E-08	0.020950508
ZNF469	R366S	23	9.54E-08	0.020950508
C20orf80	R39S	23	9.54E-08	0.020950508
FRG1B	R44S	23	9.54E-08	0.020950508
AHNAK2	M2119I	7	1.00E-07	0.0219607
SEZ6L	M430I	12	1.10E-07	0.02415677
ZNF814	D404E	37	1.11E-07	0.024376377
HLA-A	N151K	37	1.11E-07	0.024376377
TAS2R30	F185L	12	1.14E-07	0.025035198
HLA-A	G103R	8	1.15E-07	0.025254805
GIGYF2	G108R	8	1.15E-07	0.025254805
USP43	G739R	8	1.15E-07	0.025254805
NPM1	G90R	8	1.15E-07	0.025254805
OR51V1	G96R	8	1.15E-07	0.025254805
MUC4	H2685Q	21	1.15E-07	0.025254805
ZNF286B	T490S	29	1.17E-07	0.025694019
PDE4DIP	W2351*	23	1.19E-07	0.026133233
POLR2A	W326*	23	1.19E-07	0.026133233
PRAMEF19	D237E	25	1.46E-07	0.032062622
PRAMEF19	D306E	25	1.46E-07	0.032062622
GXYLT1	R126S	23	1.49E-07	0.032721443
LIPI	R442S	23	1.49E-07	0.032721443
Q8IXV1	R95S	23	1.49E-07	0.032721443
RP11-1220K2.2	F327L	15	1.53E-07	0.033599871
TAS2R43	F36L	15	1.53E-07	0.033599871
MUC4	H4221Q	15	1.53E-07	0.033599871
TP53	S215R	59	1.87E-07	0.041066509
KRTAP10-10	E28D	12	1.91E-07	0.041944937
APOBR	E361D	12	1.91E-07	0.041944937
NAF1	E64D	12	1.91E-07	0.041944937
NEB	K2613N	12	1.91E-07	0.041944937
HLA-A	K335N	12	1.91E-07	0.041944937
ASPM	K3446N	12	1.91E-07	0.041944937
PAK2	Q101H	12	1.91E-07	0.041944937
MUC4	Q2237H	12	1.91E-07	0.041944937
PCDHB8	Q639H	12	1.91E-07	0.041944937

ANKRD36C	K488N	48	1.92E-07	0.042164544
ANKRD52	E506D	22	1.93E-07	0.042384151
IRF8	E74D	22	1.93E-07	0.042384151

Supplementary Table S3. Codon usage rates of 41 amino acid alterations with a CTB

Gene	Mut AA	Mut CDS	Recur.	RSCU	ΔRSCU
TP53	R249S	AGG>AGT	537	0.90	-0.54
		AGG>AGC	31	1.44	
PIK3CA	N345K	AAT>AAA	123	0.87	-0.26
		AAT>AAG	0	1.13	
ALK	F1174L	TTC>TTA	102	0.46	-0.72
		TTC>TTG	8	0.78	
		TTC>CTC	12	1.18	
JAK2	V615L	GTA>TTA	130	0.46	0.03
		GTA>CTA	0	0.43	
KRAS	Q61H	CAA>CAC	240	1.16	0.32
		CAA>CAT	95	0.84	
SIRPA	D95E	GAC>GAG	30	1.15	0.30
		GAC>GAA	0	0.85	
SLC25A5	K296N	AAG>AAC	23	1.06	0.12
		AAG>AAT	0	0.94	
MUC4	H1309Q	CAC>CAG	28	1.47	0.94
		CAC>CAA	0	0.53	
TP53	S183*	TCA>TGA	51	1.41	0.53
		TCA>TAA	13	0.88	
LHX1	K204N	AAG>AAC	22	1.06	0.12
		AAG>AAT	0	0.94	
AGAP8	Q567H	CAG>CAC	22	1.16	0.32
		CAG>CAT	0	0.84	
ZNF429	K556N	AAG>AAC	24	1.06	0.12
		AAG>AAT	1	0.94	
JPH3	V581L	GTG>CTG	21	2.35	1.57
		GTG>TTG	0	0.78	
NBPF10	K31N	AAG>AAC	19	1.06	0.12
		AAG>AAT	0	0.94	
TP53	S106R	AGC>AGG	23	1.27	-0.02
		AGC>AGA	0	1.29	
		AGC>CGC	0	1.10	
PIK3CA	M1043I	ATG>ATT	57	1.08	0.57
		ATG>ATA	38	0.51	
		ATG>ATC	10	1.41	
TP53	K132N	AAG>AAC	82	1.06	0.12
		AAG>AAT	76	0.94	
FAM75A3	E950D	GAG>GAC	18	1.07	0.14
		GAG>GAT	0	0.93	
CCDC136	Q396H	CAG>CAC	18	1.16	0.32
		CAG>CAT	0	0.84	
SLC25A5	F271L	TTC>CTC	20	1.18	0.40
		TTC>TTA	0	0.46	
		TTC>TTG	0	0.78	
POU2F2	S449R	AGC>CGC	14	1.10	-0.19
		AGC>AGA	0	1.29	
		AGC>AGG	0	1.27	
IFITM3	G133R	GGA>CGA	11	0.66	-0.63
		GGA>AGA	0	1.29	
TP53	F134L	TTT>CTT	41	0.80	0.02
		TTT>TTA	1	0.46	
		TTT>TTG	2	0.78	
KCNJ5	G151R	GGG>AGG	169	1.27	0.06
		GGG>CGG	68	1.21	
PCLO	S1057*	TCA>TGA	20	1.41	0.53
		TCA>TAA	0	0.88	
RYBP	G291R	GGG>CGG	10	1.21	-0.06
		GGG>AGG	0	1.27	
FGFR2	W290C	TGG>TGC	15	1.09	0.18
		TGG>TGT	0	0.91	
ESYT1	S214R	AGC>AGG	18	1.27	-0.02
		AGC>AGA	0	1.29	
		AGC>CGC	0	1.10	
KMT2C	T316S	ACC>TGC	25	1.09	0.19
		ACC>AGT	1	0.90	
MLL3	T316S	ACC>TGC	25	1.09	0.19
		ACC>AGT	1	0.90	
BAT2D1	M267I	ATG>ATT	14	1.08	-0.33
		ATG>ATC	0	1.41	
		ATG>ATA	0	0.51	
STRA8	E212D	GAG>GAC	14	1.07	0.14
		GAG>GAT	0	0.93	
APOBR	E352D	GAG>GAC	14	1.07	0.14
		GAG>GAT	0	0.93	
TP53	E258D	GAA>GAT	36	0.93	-0.14
		GAA>GAC	3	1.07	
ADAD2	K16N	AAG>AAC	13	1.06	0.12
		AAG>AAT	0	0.94	
BNC2	S575R	AGT>CGT	16	0.48	-0.81
		AGT>AGA	0	1.29	
		AGT>AGG	0	1.27	
GIGYF2	G108R	GGA>CGA	8	0.66	-0.63
		GGA>AGA	0	1.29	
NPM1	G90R	GGG>CGG	8	1.21	-0.06
		GGG>AGG	0	1.27	
TP53	S215R	AGT>AGG	33	1.27	-0.02
		AGT>CGT	7	0.48	
		AGT>AGA	19	1.29	
APOBR	E361D	GAG>GAC	12	1.07	0.14
		GAG>GAT	0	0.93	
ASPM	K3446N	AAG>AAC	12	1.06	0.12
		AAG>AAT	0	0.94	
